# Research on Digital Technology Use in Cardiology: Bibliometric Analysis

**DOI:** 10.2196/36086

**Published:** 2022-05-11

**Authors:** Andy Wai Kan Yeung, Stefan Tino Kulnik, Emil D Parvanov, Anna Fassl, Fabian Eibensteiner, Sabine Völkl-Kernstock, Maria Kletecka-Pulker, Rik Crutzen, Johanna Gutenberg, Isabel Höppchen, Josef Niebauer, Jan David Smeddinck, Harald Willschke, Atanas G Atanasov

**Affiliations:** 1 Division of Oral and Maxillofacial Radiology, Applied Oral Sciences and Community Dental Care Faculty of Dentistry The University of Hong Kong Hong Kong China; 2 Ludwig Boltzmann Institute Digital Health and Patient Safety Medical University of Vienna Vienna Austria; 3 Ludwig Boltzmann Institute for Digital Health and Prevention Salzburg Austria; 4 Department of Translational Stem Cell Biology Research Institute of the Medical University of Varna Varna Bulgaria; 5 Division of Pediatric Nephrology and Gastroenterology Department of Pediatrics and Adolescent Medicine, Comprehensive Center for Pediatrics Medical University of Vienna Vienna Austria; 6 Institute for Ethics and Law in Medicine University of Vienna Vienna Austria; 7 Department of Health Promotion, Care and Public Health Research Institute Maastricht University Maastricht Netherlands; 8 Center for Human Computer Interaction Paris Lodron University Salzburg Salzburg Austria; 9 University Institute of Sports Medicine, Prevention and Rehabilitation Paracelsus Medical University Salzburg Salzburg Austria; 10 REHA Zentrum Salzburg Salzburg Austria; 11 Department of Anaesthesia, Intensive Care Medicine and Pain Medicine Medical University Vienna Vienna Austria; 12 Institute of Genetics and Animal Biotechnology of the Polish Academy of Sciences Jastrzebiec Poland

**Keywords:** cardiovascular, heart, hypertension, atrial fibrillation, cardiopulmonary resuscitation, electrocardiography, photoplethysmography, wearable device, digital health, mHealth, cardiology, cardiac, health application

## Abstract

**Background:**

Digital technology uses in cardiology have become a popular research focus in recent years. However, there has been no published bibliometric report that analyzed the corresponding academic literature in order to derive key publishing trends and characteristics of this scientific area.

**Objective:**

We used a bibliometric approach to identify and analyze the academic literature on digital technology uses in cardiology, and to unveil popular research topics, key authors, institutions, countries, and journals. We further captured the cardiovascular conditions and diagnostic tools most commonly investigated within this field.

**Methods:**

The Web of Science electronic database was queried to identify relevant papers on digital technology uses in cardiology. Publication and citation data were acquired directly from the database. Complete bibliographic data were exported to VOSviewer, a dedicated bibliometric software package, and related to the semantic content of titles, abstracts, and keywords. A term map was constructed for findings visualization.

**Results:**

The analysis was based on data from 12,529 papers. Of the top 5 most productive institutions, 4 were based in the United States. The United States was the most productive country (4224/12,529, 33.7%), followed by United Kingdom (1136/12,529, 9.1%), Germany (1067/12,529, 8.5%), China (682/12,529, 5.4%), and Italy (622/12,529, 5.0%). Cardiovascular diseases that had been frequently investigated included hypertension (152/12,529, 1.2%), atrial fibrillation (122/12,529, 1.0%), atherosclerosis (116/12,529, 0.9%), heart failure (106/12,529, 0.8%), and arterial stiffness (80/12,529, 0.6%). Recurring modalities were electrocardiography (170/12,529, 1.4%), angiography (127/12,529, 1.0%), echocardiography (127/12,529, 1.0%), digital subtraction angiography (111/12,529, 0.9%), and photoplethysmography (80/12,529, 0.6%). For a literature subset on smartphone apps and wearable devices, the Journal of Medical Internet Research (20/632, 3.2%) and other JMIR portfolio journals (51/632, 8.0%) were the major publishing venues.

**Conclusions:**

Digital technology uses in cardiology target physicians, patients, and the general public. Their functions range from assisting diagnosis, recording cardiovascular parameters, and patient education, to teaching laypersons about cardiopulmonary resuscitation. This field already has had a great impact in health care, and we anticipate continued growth.

## Introduction

### Background

Modern health care and medicine are characterized by continuous digital innovation. This innovation is driven by the confluence of, first, technological advances with transformative potential and, second, convincing use cases based on needs and opportunities from the health care domain. This is an area of high-volume activity evidenced in a large and heterogeneous scientific literature base, which warrants a high-level overview and bibliometric analysis.

### Current Transformative Developments in Digital Technology

Recent advances in digital technology for health care and medicine have been fundamentally facilitated by a revolution—increasing miniaturization and affordability—in sensing devices, which have been manufactured as both stationary and wearable devices to track a broad and growing range of vital signs and physiological measurements [1,2]. These developments have coincided with rapid innovations in interactive, networked, mobile, and ubiquitous computing [3], which has brought about modern smartphones, wireless connectivity, and Internet of Things computing, networked information systems, and increasingly capable consumer--facing and professional apps [4]. This enables effective automation in many areas that are highly relevant for health care and medicine, such as communication (eg, telehealth [5], which has been recently emphasized by an increased need for remote access for medical support in both physical and mental health during the COVID-19 pandemic [6,7]), social support [8-10], and education [11]. Moreover, there are growing possibilities for the augmentation of sensing and actuation [12], via biocompatible technologies [13] and ubiquitous sensing focused on situated functionality [14].

Technology transfer in these areas follows a general pathway from innovators and early adopters—technology developments are often inspired by hacking, gaming, or similar communities—through applied research and development into actual medical and health care practice [15]. Virtual, augmented, and mixed reality are good examples of current technologies that are beginning to take hold in real-life medical and health care practice, for example, in diagnostic and surgical procedures and rehabilitation, by offering versatility for a broad range of conditions, including pain, stroke, anxiety, depression, fear, cancer, and neurodegenerative disorders [16].

Other recent developments with transformative potential include initiatives toward digital biomarkers and interventions that promise to enable personalized and precision medicine [17]. Building on foundations developed in enthusiast communities around the quantified self [18] and personal informatics [19-21], these approaches suggest there is a need for patient data contributions and personal health records [22,23] with advances in data processing and analytics, for example, in artificial intelligence and machine learning for supporting diagnosis [24,25] and medical decision-making [26,27]. Key drivers toward truly personalized and precision medicine [28,29] will arguably be the adaptability and adaptivity of systems that anticipate rather than react [30,31], for example, via predictive modeling [32], which in turn facilitates a focus toward preventative rather than curative medicine [33].

Further potentially transformative technologies are conversational interfaces [34-36], developments that enable localized and individualized production through 3D printing [37,38], biochemical composition [39], or personal genomics [40]. These developments have considerable potential for positive change but also require delicate handling of personal data and privacy issues in accordance with data standards [41], legal and ethical considerations [42,43], and social considerations [44,45]. A key challenge lies in moving toward more sustainable adoption and use of available technologies, which requires a broad view on complex ecosystems [46,47], motivation [48] and habituation or behavior change [49-51]. Moreover, there is a need to more closely connect research and industry [52] and to work in a highly human-centered manner [53].

### Clinical Use Cases of Digital Innovation in Cardiology

The variety of digital technologies in health care and medicine is reflected in the field of cardiology, in which multiple uses can be found. Telecardiology describes the delivery of one-to-one cardiology care without the need for physical meetings between the physician and the patient [54] and has been facilitated by the improved availability and functionality of remote communication technologies and by digital technologies that enable reliable recording and transmission of clinical measurements from implantable (pacemakers, defibrillators) and consumer devices (blood pressure monitors, scales, thermometers) [54]. Cardiac telerehabilitation—programs provided at patients’ homes rather than at rehabilitation centers [55]—uses technology solutions to facilitate the remote instruction, monitoring, and supervision of patients during exercise training, with processes for providing emergency care in case of medical emergencies [56]. Artificial intelligence and machine learning approaches offer multitudes of possibilities in cardiology diagnostics and therapeutics, for example, individual cardiovascular risk factor identification; profiling, prediction, and management of cardiac arrhythmias; and enhanced cardiac imaging [17,57,58].

The field of behaviour change for primary and secondary prevention of cardiovascular disease through digital technologies, for example, to understand and modify behavior, has increased rapidly in recent years [59]. This approach could deliver effective personalized support for heart-healthy lifestyle changes, such as adherence to medication and exercise recommendations [17] with the measurement of physical activity [60] and associated parameters such as heart rate [61] using sensors incorporated in objects of daily use, such as mobile phones and watches [62]. Existing technologies also provide the ability to capture information about the environment in which behavior takes place, with mobile phone location tracking, and can be used to facilitate understanding of behavior [63] or to change behavior [64]. Behavior change interventions can be effective, especially when tailored to the individual [65]. However, there is room for improvement in terms of using the unique characteristics and full potential of digital technologies, such as the possibility of intervening at the right moment (for example when a person is in need of support). The potential of these so-called just-in-time adaptive interventions has only been explored recently, and insight into their effectiveness is largely still lacking [66].

Given that digital technologies (specifically, the internet and smartphone apps) are vehicles for information transfer, another highly promising area of application for these digital technologies in cardiology is health literacy (ie, the degree to which individuals can obtain, process, understand, and communicate about health-related information needed to make informed health decisions [67]). Health literacy is a prerequisite to successfully maintain health and self-care; navigate through the healthcare system; and in case of illness, understand health information, medication, and treatment plans [68]. Especially in older adults, health literacy is a significant predictor of information-seeking behaviors and health outcomes [68]. Despite growing global recognition of health literacy as a critical determinant of health and well-being and efforts to improve health literacy [69], health literacy levels among the global population remain low [70-72].

Digital technologies, including the internet and information communication technologies, seem to offer a convenient way to deliver broadly and rapidly evidence-based health information and thus improve overall health literacy, especially in disadvantaged populations that lack access to health care and relevant health information [73,74]. However, a recent study [75] has shown that persons with lower health literacy report difficulties searching health information and are less likely to use search engines. Moreover, low health literacy is also associated with difficulty judging the quality of health information from the internet [76]. In order to actively support individuals’ health literacy, digital technologies or services are increasingly promoted in different care contexts to accelerate patient–provider communication and, at the same time, offer an opportunity to educate patients in the appropriate use of web-based health information. In inpatient care, digital tools such as electronic displays can be employed during ward rounds to support the consultation or facilitate the discharge process, and medically vetted electronic health information is shared with patients at the hospital bedside [77]. Automated systems can be integrated to teach patients about their diagnosis and postdischarge self-care regimen [78]. In outpatient care, digital technologies often aim to support chronically ill persons. Telehealth systems for synchronous audio- and video-based communication allow patients to report symptoms and preferences to their health care provider remotely [79], while asynchronous text-based communication through patient portals enables patients more convenient access to their health information [80]. These technologies offer patients alternative modalities for information transfer and communication with health care providers, thereby facilitating effective information exchange and supporting individual health literacy skills. In the field of cardiology, the importance of a greater focus on supporting health literacy has recently been highlighted, specifically in the context of primary and secondary prevention of cardiovascular disease [81].

### Rationale for a Bibliometric Analysis

The broad range of digital technology use in cardiology is reflected by a large scientific literature base. Bibliometric analysis provides an integral view with quantitative evaluations of publishing metrics of research literature [82-84]. The purpose of this bibliometric analysis of digital technology uses in cardiology is to describe and discover current trends, topics, and scientometric characteristics within this body of literature, providing a high-level overview of the scientific literature and enabling insights for future directions in digital health in cardiology. To the best of our knowledge, no such analysis has been published to date.

## Methods

We searched the Web of Science Core Collection database on November 22, 2021 (Textbox 1).

We excluded digitalin*, digitalis*, *supplemental digital* and digital ulcer* because these words and their derivatives did not refer to digital technology, but instead referred to the drug *digitalin*, to the plant genus *Digitalis*, to supplemental digital content, and to the medical condition *digital ulcer*, respectively. No additional filters were applied to restrict the search results. The search resulted in 12,529 papers. The *Analyze Results* and *Citation Report* functions of the Web of Science platform were utilized for basic frequency counts and the number of citations per publication (mean citations per item within a subset) of the most productive authors, institutions, countries, journals, and journal categories. We also defined a subset—literature that included the terms smartphone*, *app*, or wearable* in the title, abstract, or keywords—which contained 632 papers.

The full record and cited references were then exported into VOSviewer as tab delimited files to synthesize a term map. For clarity, only terms that appeared in at least 0.5% of the literature set (>63) were included in the map. A list of top 5000 common words from the Corpus of Contemporary American English was entered to remove generic (and therefore, less meaningful) words from the term map [85]. VOSviewer was also used to identify the top 20 recurring author keywords.

As the latest digital technology uses often involve smartphone apps and wearable devices, This analysis described above, except for the term map, was similarly conducted on a subset of the concerned.

**Digital technology in cardiology search string. TS: searching for title, abstract, and keywords; WC: searching for the particular journal category.**

(#1 OR #2) NOT (#3 OR #4)

where

1. TS=(digital* AND (cardio* OR cardiac* OR heart*) NOT (digitalin* OR digitalis*))
2. WC=(CARDIAC CARDIOVASCULAR SYSTEMS) AND TS=(digital* NOT (digitalin* OR digitalis*))
3. TS=(“Supplemental Digital”)
4. TS=(“digital ulcer*”)

## Results

**Figure 1:**
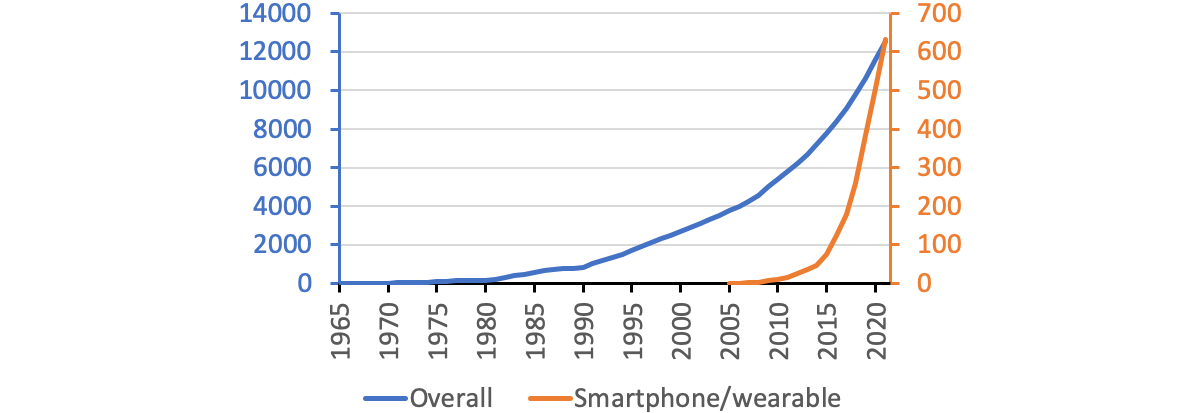
Papers published on digital technology uses in cardiology.

The 12,529 papers were published from 1965 to November 22, 2021. The earliest publication was a report on the development and demonstration of an analog-digital analyzing unit to screen heart sounds in children [86]. The literature growth seemed to be accelerating in the 2000s and especially into the 2010s (Figure 1). Approximately three-quarters of the papers (9271/12,529, 74.0%) were original articles, and review papers accounted for 6.3% (789/12,529). Proceedings papers and meeting abstracts accounted for 14.2% (1779/12,529) and 6.0% (752/12,529), respectively.

The most productive author was Professor David J Sahn from Oregon Health and Science University, whose highly cited papers were focused on real-time 3D echocardiography [87-89]. Of the 5 most productive institutions, 4 were based in the United States of America, with Harvard University having the highest number of citations per publication. The most productive journals were from the area of cardiology or cardiovascular system, with *Circulation* having the highest citations per publication among the top 5 (Table 1). *Cardiac and cardiovascular systems* was the most productive journal category, accounting for nearly one-third of the papers.

**Figure 2:**
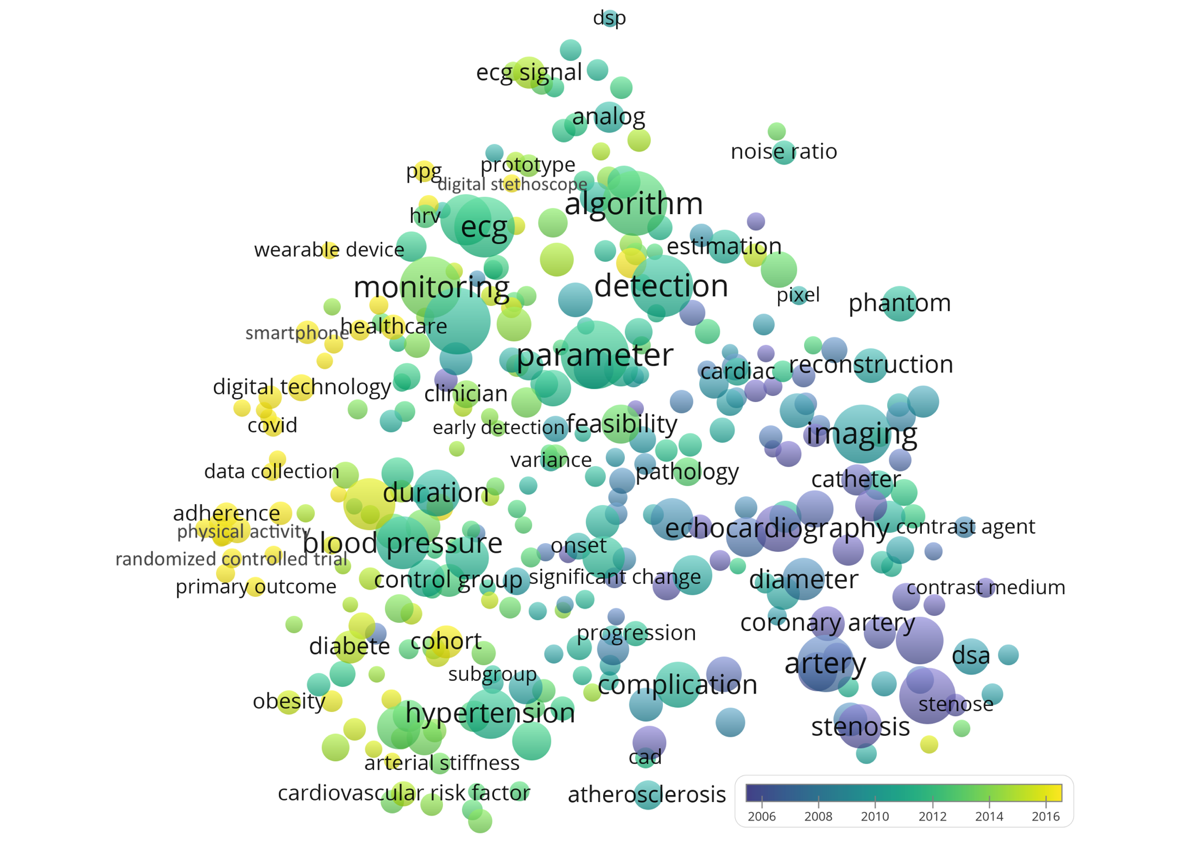
Recurring terms in the titles and abstracts of the literature about digital technology applications in cardiology. Circle size indicates publication count. Circle color indicates the average publication year. Distances between circles indicate how frequently the terms co-occurred.

The variety of digital technology uses in cardiology can be observed (Figure 2), with uses related to blood pressure (n=727, citations per publication: 20.1), hypertension (n=642, citations per publication: 21.1), arterial stiffness (n=128, citations per publication: 19.4), and stenosis (n=500, citations per publication: 23.7). Terms that appeared in more recent papers included *wearable device* (n=79, citations per publication: 10.1), *smartphone* (n=143, citations per publication: 12.0), and *COVID* (n=111, citations per publication: 3.2) (Figure 2), as well as *pandemic* (n=74, citations per publication: 3.0), *machine learning* (n=97, citations per publication: 11.1), *artificial intelligence* (n=112, citations per publication: 8.8), and *app* (n=149, citations per publication: 10.3) (not in Figure 2).

**Table 1:** Top 5 (most productive) entities in literature on digital technology use in cardiology.

Entity		N (%)	Citations per publication
**Author**			
	Sahn, David J	71 (0.5)	10.7
	Wong, Tien Yin	53 (0.5)	46.2
	Molloi, Sabee	35 (0.3)	14.9
	Jones, Molly	33 (0.3)	14.0
	Li, Xiang-Ning	33 (0.3)	4.5
**Institution**			
	University of California system	440 (3.5)	32.2
	University of London	278 (2.2)	30.8
	Harvard University	263 (2.1)	54.4
	Duke University	178 (1.4)	35.4
	US Department of Veterans Affairs	163 (1.3)	44.0
**Country**			
	United States of America	4224 (33.7)	26.7
	United Kingdom	1136 (9.1)	24.8
	Germany	1067 (8.5)	23.7
	China	682 (5.4)	12.2
	Italy	622 (5.0)	18.4
**Journal**			
	Circulation	411 (3.3)	57.4
	Journal of the American College of Cardiology	271 (2.2)	34.4
	Cardiovascular and Interventional Radiology	251 (2.0)	13.8
	American Journal of Cardiology	171 (1.4)	29.3
	European Heart Journal	140 (1.1)	20.2
**Journal category**			
	Cardiac cardiovascular systems	4101 (32.7)	22.0
	Radiology, nuclear medicine, or medical imaging	1289 (10.3)	20.9
	Peripheral vascular disease	1090 (8.7)	36.3
	Engineering, biomedical	1001 (8.0)	20.5
	Engineering, electrical or electronic	965 (7.7)	9.3

The terms *telemedicine*, *digital health*, and *mHealth (mobile health)* were among the top 20 author keywords (Table 2), suggesting that digital technology uses have been a major research focus. Such uses are also of increasing interest in the context of the COVID-19 pandemic.

The literature subset contained 632 papers on smartphone apps and wearable devices. The first paper of this subset was published in 2005, and it introduced a wearable multiparameter (including heart rate and blood pressure) ambulatory physiological monitoring system that could digitally record and continuously stream data to a base station [90]. Approximately 62.2% (393/632) of papers were original articles. Review papers accounted for 15.2% (96/632). The most productive author within this subset was Dr. Mohamed Elgendi affiliated with University of British Columbia and Simon Fraser University. His research interest focused on using data from photoplethysmography (PPG) to detect hypertension, potentially with the aid of machine learning [91,92]. Of the 5 most productive institutions, 4 were based in the United States of America. Furthermore, in the top 5 countries, the first 4 places remained unchanged from the those of the full data set (United States, United Kingdom, Germany, and China). Fifth place was taken by Australia, while Italy (ranked as the fifth most productive country in the full data set) moved to tenth place. JMIR Publications had the top 3 journals, which collectively accounted for more than 10% of the 632 papers (Table 3).

The top 20 author keywords (Table 4), notably, included *atrial fibrillation*, a cardiac condition that causes rapid and irregular heart rate.

**Table 2:** Top 20 author keywords for digital technology use in cardiology.

Author keyword	n (%)	Citations per publication
Heart rate	173 (1.4)	56.5
ECG (electrocardiography)	170 (1.4)	9.6
Telemedicine	169 (1.3)	14.8
Digital health	167 (1.3)	10.7
Blood pressure	159 (1.3)	18.4
Hypertension	152 (1.2)	17.6
Angiography	127 (1.0)	17.0
Echocardiography	127 (1.0)	38.6
Heart rate variability	126 (1.0)	18.1
Atrial fibrillation	122 (1.0)	20.6
Atherosclerosis	116 (0.9)	21.3
Cardiovascular disease	115 (0.9)	16.2
Digital subtraction angiography	111 (0.9)	12.5
Coronary artery disease	107 (0.9)	28.1
Heart failure	106 (0.8)	14.7
Machine learning	97 (0.8)	4.8
Heart	92 (0.7)	23.2
mHealth	89 (0.7)	13.7
Photoplethysmography	80 (0.6)	30.0
Arterial stiffness	80 (0.6)	22.6

**Table 3:** Top 5 in the literature subset (literature related to smartphone apps and wearable devices). The author list contains more than 5 names since multiple authors had the same number of papers.

Entity		n (%)	Citations per publication
**Author (last name, first name)**			
	Elgendi, Mohamed	9 (1.4)	18.4
	Martin, Seth S	8 (1.3)	22.5
	Sharma, Abhinav	6 (0.9)	15.3
	Ward, Rabab	6 (0.9)	23.0
	Benjamin, Emelia J.	5 (0.8)	20.2
	Majmudar, Maulik	5 (0.8)	4.0
	Marvel, Francoise A	5 (0.8)	4.0
	Murabito, Joanne M	5 (0.8)	20.2
	Shan, Rongzi	5 (0.8)	10.0
	Tarakji, Khaldoun G	5 (0.8)	42.4
	Van Hoof, Chris	5 (0.8)	13.4
**Institution**			
	University of California system	31 (4.9)	18.4
	Harvard University	27 (4.3)	10.6
	Stanford University	21 (3.3)	29.4
	University of London	18 (2.8)	14.1
	Johns Hopkins University	15 (2.4)	13.3
**Country**			
	United States of America	242 (38.3)	15.8
	United Kingdom	59 (9.3)	11.2
	Germany	51 (8.1)	7.6
	China	40 (6.3)	17.7
	Australia	37 (5.9)	8.5
**Journal**			
	JMIR mHealth and uHealth	30 (4.7)	5.8
	JMIR Research Protocols	21 (3.3)	2.3
	Journal of Medical Internet Research	20 (3.2)	11.5
	Sensors	19 (3.0)	13.1
	IEEE Access	10 (1.6)	7.8
**Journal category**			
	Engineering Electrical Electronic	135 (21.4)	8.4
	Health Care Sciences Services	104 (16.5)	8.5
	Medical Informatics	90 (14.2)	10.7
	Cardiac Cardiovascular Systems	89 (14.1)	12.0
	Engineering Biomedical	69 (10.9)	12.6

**Table 4:** Top 20 author keywords of the literature subset (literature related to smartphone apps and wearable devices).

Author keyword	n (%)	Citations per publication
Digital health	72 (11.4)	14.1
mHealth (mobile health)	47 (7.4)	12.5
Wearables	41 (6.5)	9.9
Smartphone	36 (5.7)	10.5
Telemedicine	28 (4.4)	10.0
Wearable	24 (3.8)	11.0
Heart rate	22 (3.5)	7.2
Mobile phone	21 (3.3)	8.9
Wearable devices	20 (3.2)	11.6
ECG (electrocardiography)	20 (3.2)	5.9
Physical activity	18 (2.8)	13.4
Digital medicine	17 (2.7)	18.1
Machine learning	17 (2.7)	11.9
Cardiovascular disease	16 (2.5)	14.3
Stress	16 (2.5)	3.5
Heart rate variability	15 (2.4)	6.7
Atrial fibrillation	14 (2.2)	20.9
Cardiology	14 (2.2)	4.4
Artificial intelligence	14 (2.2)	4.2
eHealth	13 (2.1)	16.1

## Discussion

Cardiovascular diseases that were frequently indicated as author keywords in the 12,529 papers included hypertension, atrial fibrillation, atherosclerosis, heart failure, and arterial stiffness. A recent meta-analysis reported that using smartphone app–based interventions could significantly lower blood pressure and improve medication adherence in patients with hypertension [93]. It was found that both wearable, ambulatory, and home monitoring devices recorded blood pressure with comparable values [94]. Smartphone and smartwatch apps could already readily distinguish atrial fibrillation from sinus rhythm and detect them with high sensitivity and specificity comparable to 12-lead electrocardiography (ECG) [95,96]. Authoritative bodies such as the European Society of Cardiology have also developed smartphone apps for patient education on atrial fibrillation [97]. The use of smartphone apps could help general physicians and trainee cardiologists decide whether a patient with heart failure should receive an implantable cardioverter defibrillator or cardiac resynchronization therapy [98]. Researchers found that these apps could potentially reduce hospital staff and facility costs by enabling patients to self-perform simple diagnostic tests, such as the 6-minute walk test, a functional exercise test used to assess patients with cardiopulmonary problems [99]. Similarly, improved access and participation in cardiac rehabilitation in terms of physical activity counselling and exercise training could be achieved by using digital health interventions that were not facility-based [100]. Apart from patient and physicians, digital technology could also target people outside of health care. For instance, massively multiplayer virtual worlds could be modified for use as a serious game to efficiently and reliably teach high school students how to perform cardiopulmonary resuscitation, an act that can be life-saving [101]. Virtual reality, a research hotspot in recent years [16], could also be utilized to teach cardiopulmonary resuscitation for medical students [102].

Meanwhile, recurring investigative modalities highlighted by the current analysis included ECG, angiography, echocardiography, digital subtraction angiography, and PPG. PPG is one of the most heavily researched diagnostic tools, and it is noninvasive, inexpensive, and convenient [103]. It could also be performed with a smartphone to detect heart rate with an average error rate as low as 1 to 1.5% [104]. Applying deep learning to PPG data could also stratify patients’ risk of hypertension [92]. Moreover, artificial intelligence could interpret ECGs rapidly with human-like performance and even detect signals and patterns largely unrecognizable by humans [105]. Overall, use cases, in which physiological parameters from wearable sensing devices are extracted and artificial intelligence is applied to draw insights, are a focal point in the literature; there is a large cluster of prominent terms such as *parameter*, *monitoring*, *detection*, and *algorithm* (Figure 2). Machine learning methods such as deep learning are frequently used to represent data structures and to make predictions or classifications, with the overall intention of supporting clinicians in data-based decision-making [106]. The expectation is that this will contribute to increasing the efficiency and effectiveness of care delivery, in particular with respect to precision health and personalized care [17]. In fact, digital technology could be very useful, with predictive models and interventions in the personalized management of cardiovascular disease patients for predicting sudden cardiac death, ventricular tachycardia, and ventricular fibrillation [107,108].

During the COVID-19 pandemic, the value of digital technology use under extreme measures for infection control has become evident. For instance, electronic stethoscopes could be utilized for contactless auscultation with real-time playback, digital storage of data, and subsequent data transmission for further assessment [109]. With the reduction of in-person hospital visits, digital technology could facilitate telemonitoring programs to serve as alternative to support patient access to care [110]. Indeed, a recent bibliometric analysis on digital health papers listed *telemedicine* and *telehealth* as two of the most frequently used keywords, indicating their relevance beyond cardiology [111].

In the subset of smartphone app and wearable device literature, we found that *mHealth*, *physical activity*, and *eHealth* were among the top author keywords, and most papers had been published in *JMIR mHealth and uHealth*, *JMIR Research Protocols*, and *Journal of Medical Internet Research*. These findings were highly consistent with a recent bibliometric analysis on digital health behavior change technology [59], but where United States, United Kingdom, and the Netherlands had been the most productive countries, in our findings, the Netherlands was replaced by Germany and China. This suggests that there are some geographical differences in research interest between cardiology-specific and general research on health behavior change. Meanwhile, another recent bibliometric analysis on mobile health apps also identified the 3 abovementioned journals as the most productive [112].

In principle, smartphone apps could offer an ideal modality for delivering digital interventions to empower patients’ self-management, by providing health literacy support and coaching content (eg, a smartphone coaching app for blood pressure control [113]). However, in line with findings from this bibliometric analysis, recent reviews [114,115] have highlighted that there is a relative paucity of health literacy interventions and, more specifically, a paucity of digital health literacy interventions for cardiovascular patient groups [116]. Moreover, apps designed to empower patients often include a narrow range of features and lack explicit linkage with theories of empowerment [117,118]. This is an area for further research—the development of content and features for such apps should be based on relevant theoretical underpinnings.

Interestingly, this bibliometric analysis did not identify top-listed terms related to *primary prevention* or *secondary prevention/cardiac rehabilitation* of cardiovascular disease. This may seem surprising, since there has been a rapid growth in the development of health apps and other digital technology interventions, and primary and secondary prevention of cardiovascular disease and cardiac rehabilitation are important areas of application [119-121]. A number of recent reviews demonstrate that a sizeable body of literature is available, for example, a systematic review and meta-analysis [122], which included 51 primary studies of digital health interventions for the primary and secondary prevention of cardiovascular disease; a systematic review and meta-analysis [123], which reported on 25 original studies of digital technology interventions for cardiovascular risk factor modification; a scoping review [124], which summarized 13 trials of mobile technology interventions for improving exercise capacity in cardiac rehabilitation; and a systematic review [100], which reported on 31 primary studies of digital health interventions for physical activity and exercise adherence in cardiac rehabilitation. In the context of this bibliometric analysis, this indicates that the literature on digital technology cardiology uses appears more accessible through disease- and condition-specific key terms (hypertension, atrial fibrillation, atherosclerosis, cardiovascular disease, and heart failure), as opposed to more service- and patient pathway–oriented terms (primary prevention, secondary prevention, and cardiac rehabilitation), which may be a relevant consideration in designing literature search strategies for researchers targeting the latter [107,108].

Another aspect of digital technology use in cardiology that was not featured prominently among the findings of this bibliometric analysis is the use of digital technology to increase the efficiency and quality of research in cardiology [125]. This refers to new possibilities afforded by mobile apps, smart devices, and implantable or wearable technologies for the design and management of research studies. Digital processes for data collection, monitoring, communication, documentation and approvals in research hold potential cost and time savings, and functionalities of digital devices open new avenues in the collection and quality control of real-time continuous data acquisition [125] (eg, real-time capture of self-reported measures and symptoms in web-based forms, and the verification of subjective data through concurrent objective measurement, for example, by supplementing subjective reports of physical activity with continuously worn activity tracking devices). The use of digital technologies in the design and management of research studies in cardiology is an emerging focus in the literature, with opportunities for robust evaluations of the advantages of digital research designs over traditional nondigital approaches.

We observed that cardiology journals were predominant in the entire literature set. The top 5 journals were also among those that had published the 100 most cited cardiovascular papers in a previous study [126], with *Circulation* and *European Heart Journal* together accounting for 64% of the top 100. However, it should be noted that digital technology use does not only involve cardiology but is an intersection between medical informatics, engineering and health sciences and services in general. With this in mind, when the literature subset on smartphone app and wearable devices was examined, it could be seen that the traditional cardiology journals have given way to newer journals that focus on digital technology and medical informatics. The *Journal of Medical Internet Research* and JMIR-portfolio journals were found to be the major publishing venues for these papers. Therefore, readers should focus not only on traditional cardiology journals when seeking the latest advancements of digital technology use in the cardiology field.

There are several limitations. First, not all journals (and hence papers) are indexed by Web of Science. Alternative databases are available, each with their own shortcomings. For example, Scopus may contain erroneous data [127], Google Scholar does not allow automated extraction of title and abstract information, and PubMed does not contain citation data. Second, publication and citation counts do not necessarily equate to scientific quality. Within the diverse cardiology research field, the baseline research productivity in particular areas could be inhomogeneous; therefore, the ranking of clinicians or researchers is given for readers’ general reference only. Notwithstanding, this study should allow readers to gain a better understanding of the literature on digital technology uses in cardiology.

Cardiovascular diseases that were frequently investigated in the literature included hypertension, atrial fibrillation, atherosclerosis, heart failure, and arterial stiffness. Recurring investigative modalities included ECG, angiography, echocardiography, digital subtraction angiography, and PPG. Readers searching for relevant information and authors searching for suitable publication venues for their work may consider that, while cardiology or cardiovascular system–focused journals were predominant in the overall literature set, the major publishing venues for the literature subset on smartphone apps and wearable devices were *Journal of Medical Internet Research* and JMIR-portfolio journals. Digital uses targeted physicians and patients as well as the general public, and their functions included assisting diagnosis, recording cardiovascular parameters, patient education, and teaching laypersons about cardiopulmonary resuscitation. The scientific body of literature on digital technology use in cardiology is rapidly growing, and its impact on health care is also expected to greatly increase in the near future.

## Abbreviations

ECG: electrocardiography
PPG: photoplethysmography
